# Early Hepatic Transcriptomic Responses to High-Fat Diet in Apolipoprotein A-IV Knockout Mice

**DOI:** 10.3390/metabo16090695

**Published:** 2026-09-20

**Authors:** Natalia Zeber-Lubecka, Maria Kulecka, Kazimiera Pyśniak, Michalina Dąbrowska, Ewa E. Hennig

**Affiliations:** 1 Department of Gastroenterology, Hepatology and Clinical Oncology, Centre of Postgraduate Medical Education, 02-781 Warsaw, Poland; natalia.zeber-lubecka@cmkp.edu.pl (N.Z.-L.);; 2 Laboratory of Cancer Metabolism, Department of Experimental Oncology, Maria Sklodowska-Curie National Research Institute of Oncology, 02-781 Warsaw, Poland

**Keywords:** Apolipoprotein A-IV, high-fat diet, whole transcriptome sequencing, gene expression profile, statin pathway, mice model

## Abstract

Background: Apolipoprotein A-IV (ApoA-IV) is involved in lipid metabolism and energy homeostasis, but its role in hepatic adaptation to dietary stress remains incompletely understood. This study aimed to determine whether ApoA-IV deficiency alters hepatic transcriptional responses to high-fat diet (HFD) and to examine how these molecular changes relate to biochemical and histological features of liver function. Methods: Male ApoA-IV knockout (KO) and wild-type (WT) mice were fed either a normal diet (ND) or HFD for 12 weeks. Liver tissues were subjected to whole-transcriptome RNA sequencing, followed by differential expression and functional enrichment analyses. Results: Histological examination revealed persistent lobular and portal inflammation accompanied by mild fibrosis in ApoA-IV-KO mice irrespective of diet, whereas steatosis and hepatocellular ballooning were absent in all groups. Both dietary intervention and genotype were associated with transcriptomic and biochemical alterations, although the identified changes were primarily evident at the level of specific genes and biological pathways. ApoA-IV deficiency was associated with a more limited set of diet-related transcriptomic changes, reflected by fewer differentially expressed genes and enriched functional categories compared to WT mice. Functional enrichment analyses identified alterations predominantly related to lipid and sterol metabolism, including the statin pathway, triglyceride metabolism, PPAR signaling and cytochrome P450-associated processes. Notably, the statin pathway was the only functional category consistently distinguishing ApoA-IV-deficient and WT mice regardless of diet. Additionally, HFD-fed ApoA-IV-KO mice exhibited elevated serum triglyceride, alanine aminotransferase and insulin levels, in contrast to WT mice. Conclusions: ApoA-IV deficiency was associated with the modulation of selected metabolic and inflammatory pathways involved in hepatic responses to dietary challenge. Cholesterol- and sterol-related pathways, including the statin pathway, represented the most consistent genotype-associated transcriptomic signature. Within the context of the present model, the findings suggest that ApoA-IV may contribute to hepatic responses during early stages of metabolic adaptation to dietary stress, although the biological significance of the observed transcriptomic differences requires further investigation.

## 1. Introduction

Apolipoprotein A-IV (ApoA-IV) is a lipid-associated protein primarily involved in lipid transport and regulation of energy homeostasis [1,2,3]. Although its systemic metabolic roles have been relatively well-described, its contribution to hepatic metabolic and inflammatory responses remains incompletely understood [4]. The liver plays a central role in the adaptation to dietary excess, particularly under high-fat diet (HFD) conditions, which typically induce metabolic stress, lipid remodeling, and inflammatory signaling [5,6]. However, the magnitude and nature of these responses are strongly influenced by genetic factors, and early transcriptional changes do not always translate directly into overt phenotypic manifestations such as steatosis [7]. Early hepatic responses to dietary excess are increasingly recognized as a dynamic and multi-layered process, where transcriptional reprogramming precedes overt morphological and metabolic alterations [8]. Exposure to HFD triggers rapid modulation of lipid metabolism, oxidative processes, and inflammatory signaling pathways, even in the absence of clear histological manifestations [9,10,11]. These early responses appear to be influenced by genetic background, suggesting that different regulatory mechanisms may shape hepatic adaptation to dietary challenge [12].

ApoA-IV is predominantly synthesized in the intestine but exerts systemic metabolic effects that influence lipid metabolism, glucose homeostasis, inflammatory responses, and gut–liver communication [13,14]. Because the aim of the present study was to investigate organism-level adaptation to dietary challenge rather than liver-autonomous mechanisms alone, a global ApoA-IV knockout model was considered the most appropriate experimental approach. While liver-specific genetic models may facilitate the investigation of tissue-specific effects, they do not fully capture the extrahepatic influences and gut–liver axis interactions that are increasingly recognized as important regulators of metabolic adaptation. Similarly, acute knockdown approaches may not reproduce the long-term compensatory mechanisms associated with lifelong ApoA-IV deficiency. Therefore, the global knockout model provides an opportunity to evaluate the integrated consequences of ApoA-IV deficiency on hepatic responses to dietary stress.

Despite emerging evidence linking ApoA-IV to metabolic regulation, it remains unclear whether ApoA-IV deficiency affects hepatic adaptation to dietary challenge, and whether its potential effects are mediated through global transcriptomic shifts or the selective modulation of specific metabolic pathways [13,15,16]. We hypothesized that ApoA-IV deficiency selectively alters hepatic transcriptional responses to HFD, affecting metabolic and stress–response pathways without inducing broad transcriptome-wide changes. In our previous study, we demonstrated that ApoA-IV deficiency modulates gut microbiota and metabolite profiles in a diet- and age-dependent manner, while limited early changes in microbiome composition were observed [17]. These findings are consistent with a contribution of host factors to the early response to dietary challenge; however, the relative contribution of host- and microbiome-dependent mechanisms remains unclear. This observation prompted us to further investigate whether ApoA-IV deficiency influences hepatic transcriptional responses, providing mechanistic insight into host adaptation to dietary stress. Therefore, in this study, we aimed to characterize the impact of ApoA-IV deficiency on hepatic gene expression profiles under normal diet (ND) and HFD conditions, and to assess how these molecular changes relate to biochemical and histological features of liver function. This study provides a framework to better understand the role of ApoA-IV in shaping hepatic metabolic and inflammatory adaptation to dietary stress.

## 2. Materials and Methods

### 2.1. Mice Model and Experiment Design

ApoA-IV knockout (KO) mice were originally generated as described by Breslow et al. [18] and maintained on a C57BL/6J genetic background. The animals used in this study were obtained from a colony provided by Professor Patrick Tso (University of Cincinnati, Cincinnati, OH, USA), as described in [17]. In the final model, exon 1 and intron 1 of the endogenous *ApoA4* gene were replaced with a neomycin resistance gene. The mice were backcrossed onto a C57BL/6J genetic background for many generations. No detectable *ApoA4* transcript and stable protein in plasma was confirmed by RNA sequencing and by Western blot analysis using ApoA4 (1D6B6) mouse antibody (Cell Signaling Technology Inc., Danvers, MA, USA), respectively.

Male ApoA-IV-KO and C57BL/6J wild-type (WT) mice were housed under temperature (21 ± 2 °C) and humidity (55 ± 10%) controlled conditions, with a 12 h light/dark cycle, and ad libitum access to food and water. Food intake was not monitored during the study, and animals were not housed in metabolic cages. Animal health, including screening for parasites, bacteria, and viruses, was monitored in accordance with the guidelines of the Federation of European Laboratory Animal Science Associations (FELASA).

During the first six weeks of life, both ApoA-IV-KO and WT mice were maintained on an ND (10% of calories from fat), containing 22% of protein, 51% of carbohydrates, 4% of fat (Labofeed H, Feed Factory Morawski, Kcynia, Poland). At six weeks of age, animals of each genotype were randomly assigned to either continue the ND or to receive an HFD (60% of calories from fat), containing 22% of protein, 25% of carbohydrates, 30% of fat (Labofeed H, Feed Factory Morawski, Kcynia, Poland), with eight mice per group. All mice were weighed individually at the start of the experiment (baseline; six weeks of age) and at the end of experiment (after an additional 12 weeks). After final weighing, mice were anesthetized with 5% isoflurane and sacrificed by cervical dislocation. The 12-week dietary intervention was selected to induce diet-associated metabolic alterations and enable assessment of hepatic, biochemical, and transcriptomic responses to HFD exposure. Samples of blood and liver tissue were immediately collected and stored at −80 °C until use.

### 2.2. Histological Evaluation

Fragments of fresh liver tissue measuring 1 cm^2^ were washed with saline, immediately 4% paraformaldehyde-fixed, and stained with hematoxylin and eosin for histological evaluation. Histological features, including lobular and portal inflammation, hepatocyte ballooning, steatosis, and fibrosis, were scored according to the combined metabolic dysfunction-associated steatotic liver disease (MASLD) activity score system [19,20]. To facilitate visualization of collagen deposition, selected liver sections were additionally stained with Masson’s trichrome stain.

### 2.3. Serum Biochemical Parameters

Serum concentrations of total cholesterol, high-density lipoprotein cholesterol (HDL-C), triglycerides, alanine aminotransferase (ALT), aspartate aminotransferase (AST), alkaline phosphatase (ALP), and glucose were measured using a Spotchem EZ SP-4430 analyzer (ARKRAY, Kyoto, Japan) with dedicated reagent strips, according to the manufacturer’s instructions. Serum insulin concentration was measured by a rat/mouse insulin ELISA test (Merck Millipore, Burlington, MA, USA).

### 2.4. Total RNA Extraction, Library Preparation, and Whole Transcriptome Sequencing

Total RNA was extracted from frozen liver tissues using a Qiagen RNA isolation kit (Qiagen, Hilden, Germany), according to the manufacturer’s instructions. RNA concentration and purity were assessed using a NanoDrop spectrophotometer (Thermo Fisher Scientific, Waltham, MA, USA), based on the A260/280 and A260/230 ratios, respectively. RNA integrity number (RIN) and distribution value 200 (DV200) were evaluated using the Agilent RNA 6000 Nano Kit on a 2100 Bioanalyzer (Agilent Technologies, Santa Clara, CA, USA). Only RNA samples with RIN ≥ 8 were included in downstream library preparation. RNA samples were stored at −80 °C until further processing.

Whole transcriptome libraries were prepared from poly(A)-selected RNA using the Ion Total RNA-Seq Kit v2 (Thermo Fisher Scientific, Waltham, MA, USA), following the manufacturer’s protocol. Poly(A) enrichment was used for library preparation; therefore, the analysis primarily targeted polyadenylated transcripts and was not intended for comprehensive characterization of non-coding RNAs lacking poly(A) tails. The workflow included first- and second-strand cDNA synthesis and the preparation of strand-specific sequencing libraries. Template preparation and enrichment were performed using the Ion Chef System (Thermo Fisher Scientific, Waltham, MA, USA) with the Ion PI™ Hi-Q™ Chef Kit, according to the manufacturer’s instructions. Sequencing was carried out on the Ion Proton platform (Thermo Fisher Scientific, Waltham, MA, USA) using Ion PI™ Chip v3 and the Ion PI™ Hi-Q™ Sequencing Kit, following the manufacturer’s protocols.

Samples failing predefined quality-control criteria (RNA integrity requirements and/or sequencing quality metrics) were excluded from downstream analyses. The final number of biological replicates included in each comparison is provided in the Results section.

### 2.5. Data and Statistical Analyses

The statistical differences in weight gain and biochemical parameters were analyzed by Kruskal–Wallis test, followed by FDR-corrected [21] Mann–Whitney U test; *p*- or FDR-adjusted *p*-values less than 0.05 were considered significant, respectively.

For whole transcriptome sequencing (WTS) data analysis, raw reads were mapped to mm39 reference genome with STAR [22] version 2.7.10 as the primary aligner, and bowtie2 [23] version 2.4.4 for previously unaligned reads. For bowtie2 the running mode was—*very-sensitive-local*. Transcript abundance (on a gene level) was quantified with htseq-count version 2.0.9 (HTSeq framework [24]) using Ensembl Gene gtf file from UCSC as reference and “union” mode as an option for transcript quantification. Differential transcript abundance was assessed using DESeq2 version 1.38.3 with default parameters. For genes with base mean expression higher than 5, *p*-values were adjusted (*p*_adj_) for multiple testing using the Benjamini–Hochberg algorithm. *P*_adj_-values less than 0.05 were considered significant. Plots were made in R software using ggplot2 and EnhancedVolcano [25] version 1.16 packages. The principal component analysis (PCA) was performed on transcriptomic data, and statistical significance of the related groupings was assessed with PERMANOVA using adonis2 function from vegan package [26] version 2.6.

Functional enrichment analysis of differentially expressed genes (DEGs) was performed using the Cytoscape platform (version 3.6.1) with the ClueGO plugin (version 2.5.1), focusing on Gene Ontology (GO) categories, including Biological Processes (BP), Molecular Function (MF), Cellular Component (CC) and Immune System Process (ISP), as well as Reactome Pathways, Wiki Pathways and KEGG categories. Default parameters were applied, and *p*-values were adjusted using the Benjamini–Hochberg method, with a significance threshold set at 0.05.

## 3. Results

A mouse model was used to evaluate the possible role of ApoA-IV in early metabolic adaptation to dietary stress. In groups of ApoA-IV-KO and WT mice, fed either an ND or HFD for 12 weeks, body weight gain, serum biochemical parameters, and liver histological characteristics were evaluated. Finally, liver RNA sequencing (RNA-seq) profiles were compared between groups to assess the effect of ApoA-IV deficiency on gene expression.

### 3.1. Mice Characteristics

**Figure 1:**
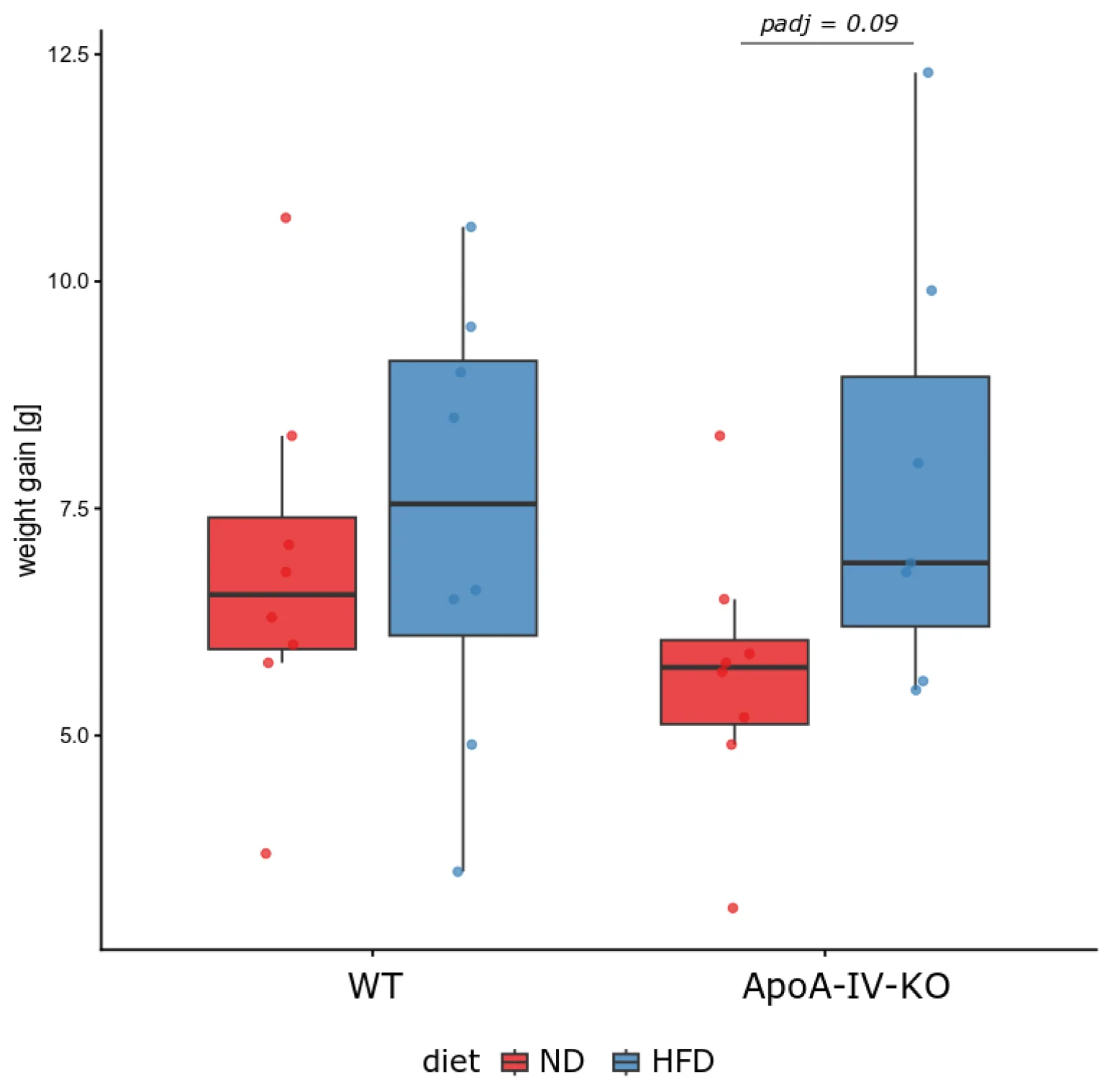
Body weight gain in WT and ApoA-IV-KO mice following 12 weeks of dietary intervention. Data are presented as box-and-whisker plots showing the median (center line), interquartile range (box), and 1.5 × IQR distance from 1st and 3rd quartile (whiskers). Individual animals are shown as red or blue dots. FDR-adjusted *p*-value obtained using the Mann–Whitney U test is indicated. ApoA-IV-KO, ApoA-IV knockout; HFD, high-fat diet; ND, normal diet; WT, wild-type.

Each mouse was individually weighed at the baseline and at the end of the experiment. No statistically significant differences (FDR-adjusted *p*-value > 0.05) were observed in median body weight gain between HFD- and ND-fed mice of both genotypes, as well as between mice of both genotypes regardless of diet (Figure 1). However, numerically greater weight gain was observed in mice fed an HFD, particularly in those with ApoA-IV deficiency (FDR-adjusted *p* = 0.09).

The diet had a rather subtle effect on the histological appearance of the liver in the individual mouse strains, considering the mean score values (Table 1 and Figure S1). At the end of the experiment, the livers of WT mice were histologically normal. Furthermore, no ballooned hepatocyte or steatosis was observed in any of the animal groups. Lobular and portal inflammation, which reached histological grades 1–3, was present in all but one of the ApoA-IV-KO mice, regardless of diet, and in most of the WT mice on the HFD, especially in portal areas. The inflammation was accompanied by mild fibrosis, which was present at grade 1 or 2 in all ApoA-IV-deficient mice and half of the WT mice.

Table 2 presents the medians of baseline serum concentration of biochemical parameters for both strains and diets. Pairwise comparisons showed that feeding the HFD was associated with significantly (FDR-adjusted *p*-value < 0.05) higher serum levels of total cholesterol, HDL-C, and lower levels of ALP, compared to ND feeding, regardless of the mouse strain (Figure S2). In addition, ApoA-IV-deficient mice on the HFD exhibited elevated levels of triglycerides, ALT, and insulin. Notably, HFD-fed ApoA-IV-KO mice had lower total cholesterol levels than those of the WT mice. In contrast, insulin levels were higher on the HFD and lower on the ND compared to the WT mice. Serum glucose concentrations did not differ significantly between the groups (Table 2 and Figure S2).

### 3.2. Differentially Expressed Genes

WTS of total RNA samples was used to determine gene expression profiles in liver tissues from ApoA-IV-KO and WT mice, fed an HFD or ND, with six mice in ApoA-IV-KO/ND group, three mice in ApoA-IV-KO/HFD group, eight mice in WT/ND group and five mice in WT/HFD group in final analysis. An average of 21,761,513 reads (SD = 7,010,861) was generated per sample, with an average of 96% of reads mapping to the mm39 genome (SD = 1%). The average read length was 225 bp (SD = 98 bp), and on average, 69% of mapped reads fell within exonic regions (SD = 4%), while the remaining reads were either intronic (3%), intergenic (8%), or spanned multiple types of regions. Noticeable expression (i.e., with a base mean for genes with at least 5 reads in total in at least one compared group) was shown for 12715 genes in ND-fed ApoA-IV-KO mice samples, and for 12485 and 12836 genes in HFD-fed and ND-fed WT mice samples, respectively. As shown in Figure S3, PCA incorporating gene expression data for all study groups did not reveal any significant clustering of samples (PERMANOVA: R^2^ = 0.177 and *p* = 0.237).

In pairwise comparisons between ApoA-IV-KO and WT mice, 50 and 19 DEGs (*p_adj_* < 0.05) were indicated for ND-fed and HFD-fed animals, respectively (presented in Tables S1 and S2, respectively). Of these, 25 (50%) and 5 (26.3%) genes were more highly expressed in ApoA-IV-KO mice fed an ND and HFD, respectively. In turn, the HFD vs. ND comparison identified 16 DEGs in liver tissues of ApoA-IV-deficient mice and 45 DEGs in WT mice (presented in Tables S3 and S4, respectively). Of these, 7 (38.9%) genes were up-regulated on the HFD in ApoA-IV-KO mice and 9 (20%) genes in WT mice.

**Figure 2:**
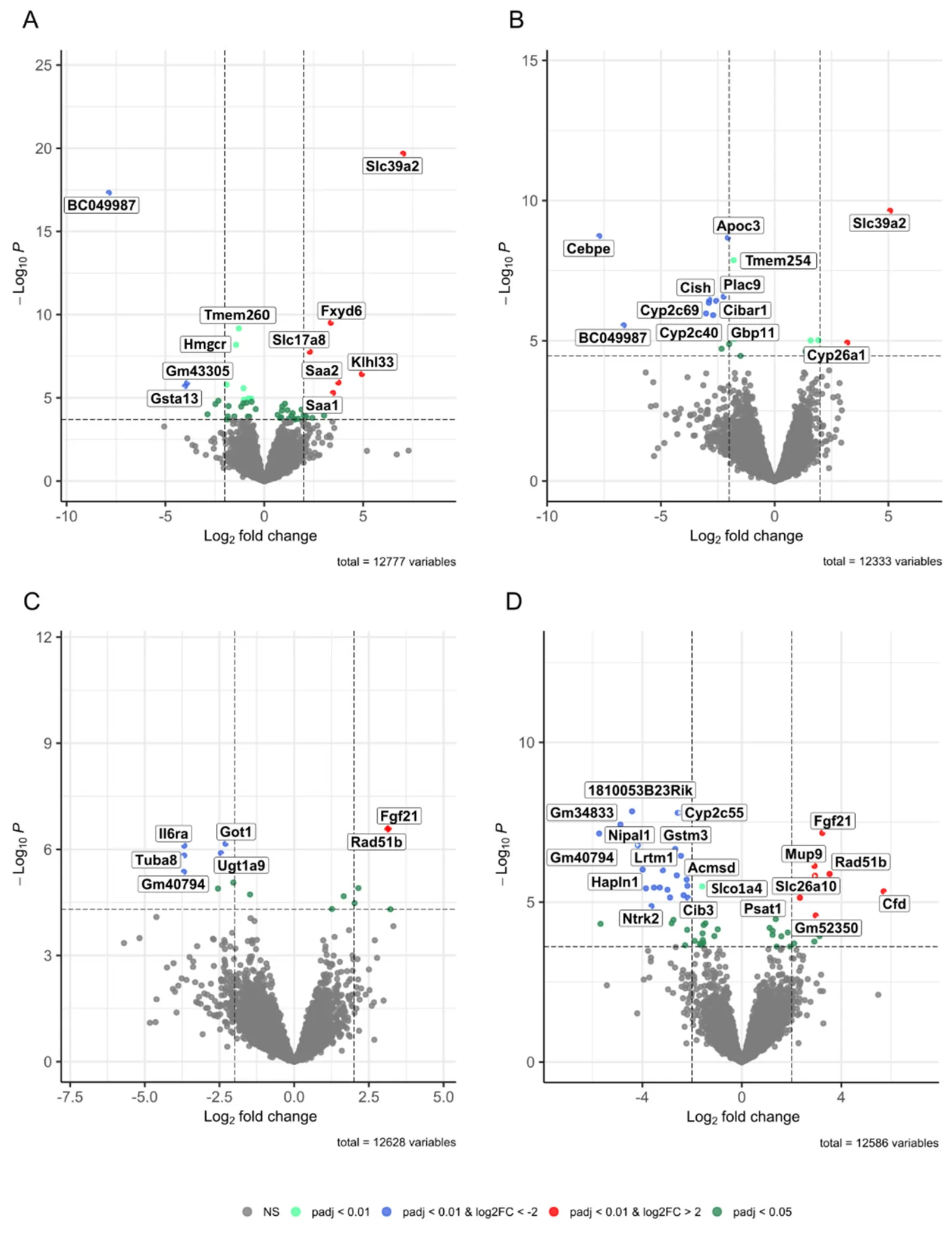
Volcano plots showing significant differentially expressed genes (DEGs) from comparisons: (**A**) ApoA-IV-KO vs. WT ND-fed mice, (**B**) ApoA-IV-KO vs. WT HFD-fed mice, (**C**) HFD-fed vs. ND-fed ApoA-IV-KO mice, and (**D**) HFD-fed vs. ND-fed WT mice. ApoA-IV-KO, ApoA-IV knockout; HFD, high-fat diet; ND, normal diet; WT, wild-type.

Volcano plots were generated to visualize the distribution of significant DEGs across all pairwise comparisons (Figure 2). The top five DEGs most significantly differentiating between ApoA-IV-KO and WT mice liver tissues included *Slc39a2* (log_2_FC = 7.16; *p*_adj_ = 1.12 × 10^−17^), *BC049987* (log_2_FC = −7.84; *p*_adj_ = 1.22 × 10^−14^), *Tmem260* (log_2_FC = −1.28; *p*_adj_ = 1.55 × 10^−07^), *Hmgcr* (log_2_FC = −1.48; *p*_adj_ = 8.97 × 10^−07^) and *Slc17a8* (log_2_FC = 2.29; *p*_adj_ = 4.28 × 10^−06^) in samples from ND-fed mice and *Slc39a2* (log_2_FC = 5.08; *p*_adj_ = 2.85 × 10^−06^), *Cebpe* (log_2_FC = −7.71; *p*_adj_ = 8.68 × 10^−06^), *ApoC3* (log_2_FC = −2.06; *p*_adj_ = 8.68 × 10^−06^), *Tmem254* (log_2_FC = −1.8; *p*_adj_ = 4.10 × 10^−05^) and *Plac9* (log_2_FC = −2.24; *p*_adj_ = 6.57 × 10^−04^) in samples from HFD-fed mice. While the most significant DEGs in HFD vs. ND included *Fgf21* (log_2_FC = 3.17; *p*_adj_ = 8.75 × 10^−04^), *Rad51b* (log_2_FC = 3.11; *p*_adj_ = 8.75 × 10^−04^), *Il6ra* (log_2_FC = −3.68; *p*_adj_ = 1.65 × 10^−03^), *Got1* (log_2_FC = −2.3; *p*_adj_ = 1.65 × 10^−03^) and *Ugt1a9* (log_2_FC = −2.45; *p*_adj_ = 2.31 × 10^−03^) in samples from ApoA-IV-deficient mice, and *Cyp2c55* (log_2_FC = −2.51; *p*_adj_ = 6.75 × 10^−05^), *Fgf21* (log_2_FC = 3.3; *p*_adj_ = 6.75 × 10^−05^), *Gm40794* (log_2_FC = −5.63; *p*_adj_ = 8.32 × 10^−05^), *Slco1a4* (log_2_FC = −1.47; *p*_adj_ = 1.78 × 10^−04^) and *1810053823Rik* (log_2_FC = −4.26; *p*_adj_ = 1.78 × 10^−04^) in WT mice samples (Figure 2 and Tables S1–S4).

### 3.3. Functional Enrichment Analyses

For functional enrichment analyses, the significance threshold was relaxed from *p*_adj_ < 0.05 to *p*_adj_ < 0.1 to include a broader set of potentially relevant genes and improve pathway-level interpretation. Due to the limited number of DEGs identified under the primary significance threshold, a more inclusive threshold was additionally explored to facilitate functional enrichment analysis and biological interpretation at the pathway level. Gene Set Enrichment Analysis (GSEA) was not performed in the present study. Genes included in these analyses are listed in Tables S1–S4.

**Figure 3:**
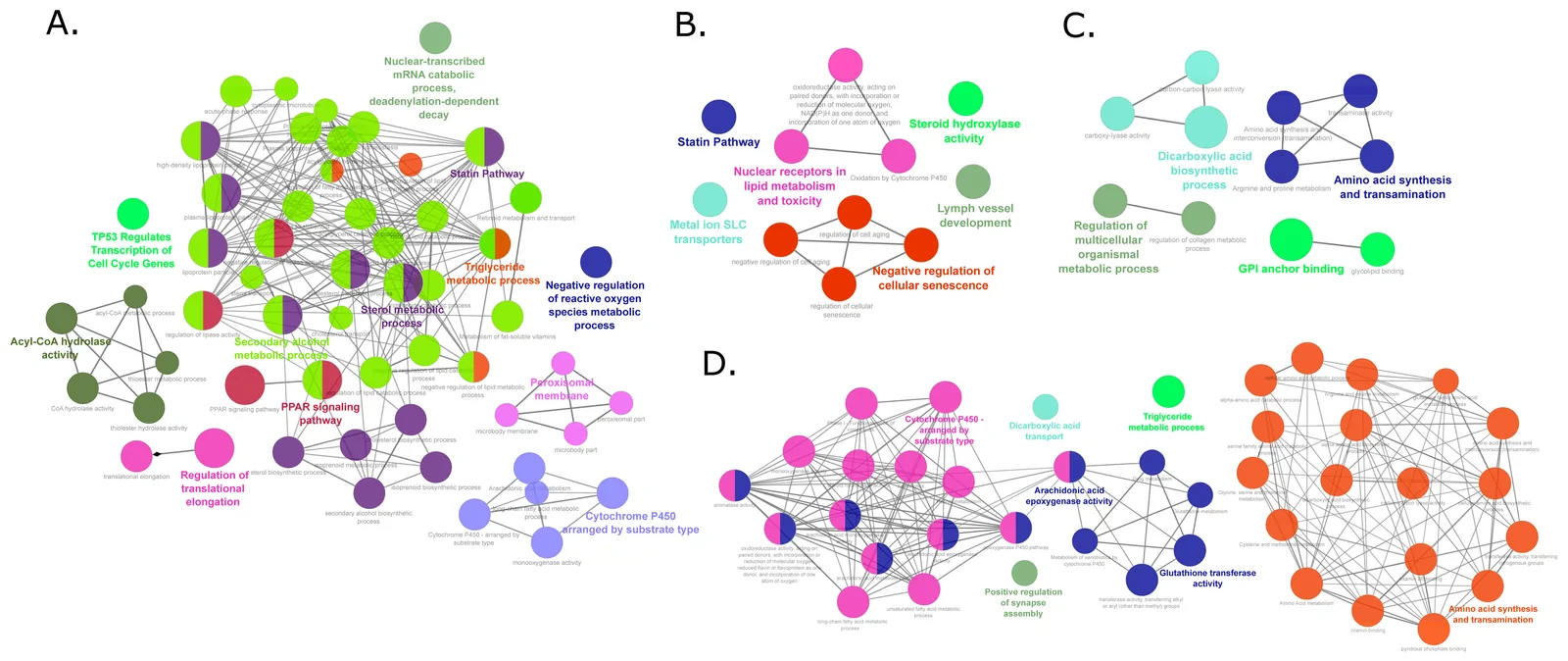
ClueGo functional enrichment analysis for significant differentially expressed gene sets from comparisons: (**A**) ApoA-IV-KO vs. WT ND-fed mice, (**B**) ApoA-IV-KO vs. WT HFD-fed mice, (**C**) HFD-fed vs. ND-fed ApoA-IV-KO mice, and (**D**) HFD-fed vs. ND-fed WT mice. Statistical significance: *p*_adj_ < 0.05. ApoA-IV-KO, ApoA-IV knockout; HFD, high-fat diet; ND, normal diet; WT, wild-type.

The functional annotations of these gene sets were analyzed using the ClueGO tool based on categories from the GO database (BP, MF, CC and ISP terms), KEGG, Reactome Pathways, and Wiki Pathways. ClueGO analysis revealed that 124 DEGs identified in the comparison between ND-fed ApoA-IV-KO and WT mice significantly (*p*_adj_ < 0.05) enriched terms that centered around *Secondary alcohol metabolic process*, *Sterol metabolic process*, *Statin pathway*, *Triglyceride metabolic process*, *PPAR signaling pathway*, *Acyl-CoA hydrolase activity* and *Cytochrome P450—arranged by substrate type* (Figure 3A and Table S5). In turn, only 11 pathways were enriched by 32 DEGs that differentiated ApoA-IV-deficient and WT mice on the HFD, including *Nuclear receptors in lipid metabolism and toxicity*, *Negative regulation of cellular senescence* and *Statin pathway* (Figure 3B and Table S6).

Similarly, 11 functional categories were enriched with 20 DEGs from the comparison of ApoA-IV-deficient mice on the HFD and those on the ND, grouped into: *Amino acid synthesis and transamination*, *Dicarboxylic acid biosynthetic process*, *GPI anchor binding* and *Regulation of multicellular organismal metabolic process* (Figure 3C and Table S7). In turn, 65 DEGs from the HFD vs. ND comparison for WT mice were annotated to pathways associated with *Amino acid synthesis and transamination*, *Cytochrome P450—arranged by substrate type, Arachidonic acid epoxygenase activity*, *Glutathione transferase activity* and *Triglyceride metabolic process* (Figure 3D and Table S8).

**Figure 4:**
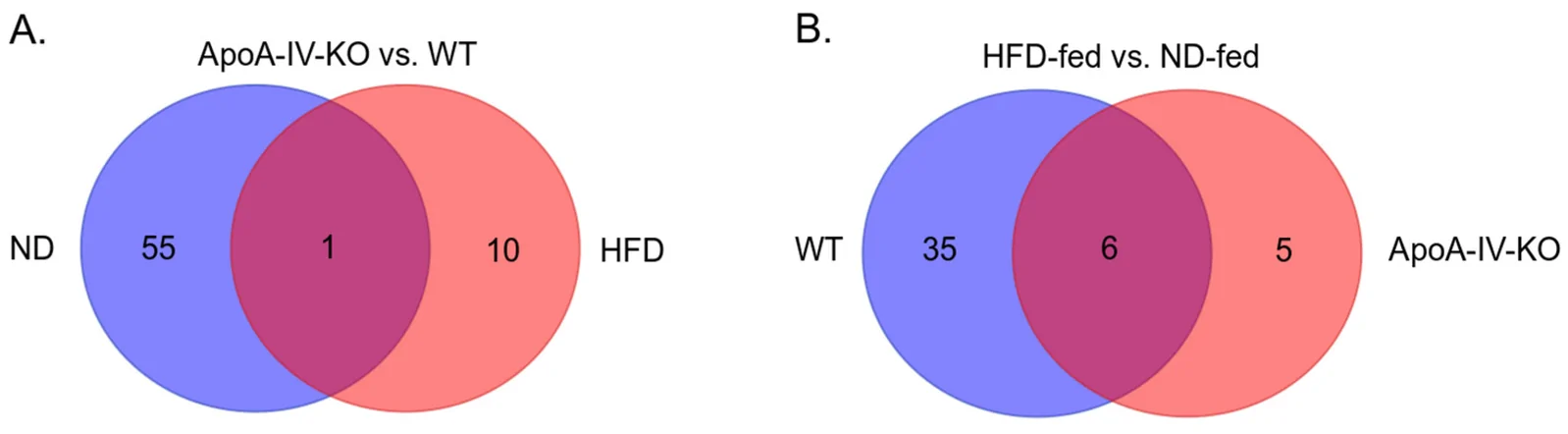
Venn diagrams illustrating shared and genotype- or diet-specific functional categories in ApoA-IV-KO vs. WT mice (**A**) and in HFD-fed vs. ND-fed mice (**B**). ApoA-IV-KO, ApoA-IV knockout; HFD, high-fat diet; ND, normal diet; WT, wild-type.

As shown in Figure 4A, 55 functional categories enriched by DEGs identified in the ApoA-IV-KO vs. WT comparison were unique to mice on the ND and only 10 were unique to mice on the HFD, such as *Oxidation by Cytochrome P450*, *Steroid hydroxylase activity*, *Nuclear receptors in lipid metabolism and toxicity* and *Negative regulation of cellular senescence* (Table S9). However, the *Statin pathway* was the only pathway that differentiated ApoA-IV-deficient and WT mice, regardless of diet. The genes contributing to the *Statin pathway* enrichment included *ApoC3* (log_2_FC = −1.64; *p*_adj_ = 0.0211), *Hmgcr* (log_2_FC = −1.48; *p*_adj_ = 8.97 × 10^−07^), *Lipc* (log_2_FC = 0.80; *p*_adj_ = 0.0193), and *Lrp1* (log_2_FC = 1.418; *p*_adj_ = 0.099) in the ApoA-IV-KO vs. WT comparison under ND conditions, whereas under HFD conditions the pathway was represented primarily by *ApoC3* (log_2_FC = −2.058; *p*_adj_ = 2.11 × 10^−09^) and *Cyp7a1* (log_2_FC = −1.998; *p*_adj_ = 0.01).

When comparing HFD-fed and ND-fed mice, 35 functional categories were unique to WT mice, while only five were unique to ApoA-IV-KO mice, such as *Regulation of multicellular organismal metabolic process*, *GPI anchor binding,* and *Glycolipid binding* (Figure 4B and Table S10). Six pathways were shared by both strains, including *Amino acid synthesis and transamination*, *Arginine and proline metabolism* and *Dicarboxylic acid biosynthetic process*.

## 4. Discussion

In this study, we demonstrate that ApoA-IV deficiency does not induce broad transcriptome-wide changes in the liver but instead leads to selective alterations affecting defined metabolic and stress–response pathways. This pattern is consistent with a modulatory role of ApoA-IV in hepatic adaptation rather than a primary driver of global transcriptional reprogramming. Although no global transcriptomic separation between groups was observed, both dietary intervention and ApoA-IV deficiency were associated with distinct transcriptomic and biochemical alterations. These differences were primarily evident at the level of specific genes and biological pathways rather than broad transcriptome-wide changes. ApoA-IV-deficient mice exhibited a smaller number of diet-associated DEGs than WT animals. Given the limited sample size and modest transcriptomic effect sizes, these observations should be interpreted cautiously and may reflect subtle differences in hepatic adaptation to dietary challenge. Noteworthy was the presence of inflammatory and mild fibrotic lesions in ApoA-IV-deficient mice even under normal dietary conditions, consistent with the presence of a baseline pro-inflammatory hepatic phenotype. This interpretation is consistent with previous studies demonstrating that ApoA-IV participates in metabolic responses to nutrient excess and regulates pathways involved in hepatic lipid metabolism, insulin sensitivity and adaptation to high-fat feeding [27,28].

The lack of significant global separation in PCA suggests that neither genotype nor diet accounted for a large proportion of the overall transcriptomic variance in the studied groups. However, the absence of clear clustering does not preclude biologically meaningful changes affecting a limited subset of genes and pathways. The present findings therefore suggest that ApoA-IV deficiency and dietary intervention were associated with relatively subtle transcriptomic alterations rather than extensive transcriptional reprogramming. In addition, inter-individual variability and the limited number of biological replicates available for some comparisons may have reduced the power to detect broader transcriptomic patterns at the PCA level.

This pattern was also reflected at the level of serum biochemical parameters, where both dietary intervention and genotype were associated with measurable metabolic alterations. Similar observations are consistent with previous studies showing that ApoA-IV deficiency alters selected pathways involved in glucose and lipid metabolism without inducing profound disturbances in systemic glucose homeostasis under basal conditions [1]. Consistent with these observations, serum glucose levels did not differ significantly between groups in the present study, suggesting that the observed metabolic alterations occur without overt impairment of glucose homeostasis.

The transcriptional response to dietary intervention differed markedly between genotypes, with WT mice exhibiting a broader range of DEGs compared to ApoA-IV-deficient animals. However, this observation should be interpreted with caution. The number of biological replicates available for the ApoA-IV/HFD group was limited and may have reduced statistical power, thereby affecting the number of detected DEGs. Moreover, genotype-dependent differences were also observed under normal dietary conditions, where the comparison between ApoA-IV-deficient and WT mice yielded a larger number of DEGs than the diet-based comparison within ApoA-IV-deficient animals.

In addition to differences in the magnitude of response, genotype-dependent patterns were observed at the pathway level, with WT mice showing enrichment in lipid metabolism and detoxification pathways, whereas ApoA-IV-deficient mice exhibited a more limited response characterized by a distinct pattern of pathway enrichment. The markedly reduced number of DEGs and enriched functional categories observed in ApoA-IV-deficient mice suggests that transcriptomic responses to dietary intervention differed between genotypes. The biological significance of these genotype-dependent differences remains uncertain and will require confirmation in future mechanistic studies. Adaptive transcriptional responses to nutritional challenges are largely coordinated by metabolic regulators such as PPARs, which adjust hepatic lipid utilization and energy metabolism according to nutrient availability [29,30]. In this context, the observed pathway-level differences may be consistent with a role of ApoA-IV in processes involved in maintaining hepatic homeostasis under conditions of nutrient excess.

Functional enrichment analyses predominantly identified pathways related to lipid and sterol metabolism, including the statin pathway, cytochrome P450-dependent processes, and triglyceride metabolism. Similar observations have been reported in ApoA-IV-deficient rats, where liver RNA-seq revealed alterations in genes involved in glycolysis, gluconeogenesis and de novo lipogenesis, supporting a broader role of ApoA-IV in coordinating hepatic metabolic pathways [1]. The statin pathway, reflecting cholesterol biosynthesis and its regulation, was the only functional category differentiating ApoA-IV-deficient and WT mice irrespective of diet, supporting the suggestion of a stable genotype-dependent effect on hepatic cholesterol handling [31]. This observation suggests that ApoA-IV deficiency may exert a stable effect on hepatic sterol metabolism that is detectable even in the absence of dietary challenge. The enrichment of this pathway was associated with differential expression of *Hmgcr*, the rate-limiting enzyme of cholesterol biosynthesis, together with additional genes involved in cholesterol homeostasis and lipoprotein metabolism, including *Cyp7a1*, *Lrp1, Lipc*, and *ApoC3*. The observed downregulation of *Hmgcr* and *Cyp7a1*, accompanied by altered expression of genes involved in lipoprotein uptake and remodeling (*Lrp1* and *Lipc*), suggests that ApoA-IV deficiency may influence multiple interconnected components of hepatic cholesterol handling rather than a single isolated pathway [32]. Furthermore, the reduced expression of *ApoC3*, despite elevated circulating triglyceride concentrations, highlights the complexity of the metabolic phenotype and suggests that changes in triglyceride homeostasis are likely driven by multiple regulatory mechanisms [33]. Given the central role of cholesterol homeostasis in regulating hepatic lipid metabolism and metabolic adaptation, these findings suggest that altered sterol biosynthesis may represent one of the most robust molecular consequences of ApoA-IV deficiency. HMGCR is under the control of the SREBP2 regulatory network, which plays a central role in maintaining hepatic cholesterol homeostasis [34]. Although the present data do not allow direct assessment of SREBP2 activity, the persistent enrichment of cholesterol biosynthesis-related genes may be consistent with altered sterol-sensing mechanisms or compensatory regulation aimed at maintaining intracellular cholesterol balance. The involvement of genes associated with both cholesterol biosynthesis and lipoprotein metabolism may also help explain why alterations within this pathway were accompanied by changes in circulating lipid parameters, suggesting that the cholesterol biosynthesis signature identified in ApoA-IV-deficient mice may have functional metabolic consequences [35]. Further mechanistic studies will be required to determine whether ApoA-IV deficiency influences cholesterol homeostasis through SREBP2-dependent pathways.

Previous studies have similarly demonstrated that ApoA-IV deficiency promotes hepatic lipogenesis and insulin resistance, whereas ApoA-IV overexpression suppresses lipogenic programs and improves hepatic insulin signaling, highlighting its role in metabolic regulation [27]. This finding supports previous studies indicating that cholesterol biosynthesis pathways are tightly regulated in response to metabolic stress and play a central role in hepatic adaptation to dietary lipid excess [36]. PPAR-related pathways were also enriched, in agreement with the established role of PPAR transcription factors in regulating lipid utilization, fatty acid oxidation and adaptation to nutritional stress [30,37]. As key regulators of processes associated with metabolic flexibility, PPAR-dependent pathways enable dynamic adjustment of cellular energy metabolism in response to nutrient availability [38]. In parallel, the repeated enrichment of cytochrome P450-related pathways may suggest an association between ApoA-IV deficiency and pathways involved in lipid oxidation and xenobiotic metabolism [39].

Several of the most strongly affected genes provide additional biological context for the enriched pathways. Among them, *Fgf21* was consistently upregulated in response to HFD in both genotypes, suggesting activation of a conserved adaptive response to lipid overload [40]. FGF21 is a well-established metabolic regulator induced during fasting, ketogenic conditions and high-fat feeding, where it promotes fatty acid oxidation and metabolic adaptation [41]. The pronounced induction of *Fgf21* in both genotypes may therefore represent an early compensatory mechanism aimed at maintaining hepatic metabolic homeostasis during lipid excess. In contrast, *Hmgcr*, encoding the rate-limiting enzyme of cholesterol biosynthesis [42], was differentially expressed between ApoA-IV-deficient and WT mice under ND conditions, supporting the prominent enrichment of cholesterol- and sterol-related pathways. Likewise, reduced expression of *ApoC3* in ApoA-IV-deficient animals is compatible with alterations in lipid metabolism [1], as ApoC3 is a key regulator of triglyceride-rich lipoprotein metabolism [43]. Despite the expectation that reduced *ApoC3* expression would favor triglyceride clearance, ApoA-IV-deficient mice displayed elevated circulating triglyceride levels under HFD, suggesting that ApoA-IV deficiency affects additional pathways involved in triglyceride homeostasis beyond ApoC3-dependent regulation [44]. ApoA-IV has been implicated in multiple aspects of triglyceride metabolism, including chylomicron assembly and metabolism, triglyceride-rich lipoprotein clearance, and broader regulation of systemic lipid homeostasis [45]. Therefore, the elevated triglyceride concentrations observed in ApoA-IV-deficient mice may reflect the combined effects of multiple metabolic disturbances rather than isolated ApoC3-dependent mechanisms [14]. In this context, alterations in lipoprotein metabolism and triglyceride turnover may contribute to the observed phenotype.

*Slc39a2* was among the most strongly affected genes and exhibited the largest expression difference between genotypes under both dietary conditions. Given the reported links between zinc homeostasis, inflammatory signaling and tissue remodeling, altered *Slc39a2* expression may warrant further investigation in the context of the inflammatory and fibrotic phenotype observed in ApoA-IV-deficient mice [46]. Although the functional significance of these individual genes requires further investigation, their biological roles are broadly consistent with the pathway-level changes identified in the present study. These gene-level alterations suggest that ApoA-IV deficiency primarily affects pathways involved in cholesterol handling, lipid transport, metabolic stress responses and inflammatory adaptation rather than inducing widespread transcriptional dysregulation [45].

We observed the consistent presence of lobular and portal inflammation accompanied by mild fibrosis in ApoA-IV-deficient mice, irrespective of dietary intervention. Previous single-cell transcriptomic analyses demonstrated that ApoA-IV deficiency promotes pro-inflammatory remodeling of the hepatic immune compartment, including expansion of inflammatory macrophage and granulocyte populations [47]. Particularly noteworthy was the presence of these inflammatory changes even under normal dietary conditions, which may suggest that ApoA-IV deficiency is associated with a baseline pro-inflammatory hepatic phenotype that precedes exposure to dietary stress [48]. Such pre-existing low-grade inflammation could contribute to the hepatic phenotype observed following HFD exposure and may influence subsequent adaptive responses to metabolic challenge [49]. Previous studies have also reported anti-inflammatory and metabolically protective functions of ApoA-IV, supporting the possibility that its deficiency may increase susceptibility to inflammatory responses under both physiological and metabolically challenging conditions [14,45]. In our model, inflammatory and fibrotic changes were among the most prominent histological alterations despite the generally mild hepatic phenotype, suggesting that ApoA-IV may exert protective functions extending beyond the regulation of lipid storage. Although the underlying mechanisms remain unclear, these observations are consistent with a role for ApoA-IV in maintaining hepatic homeostasis under both physiological and metabolically challenging conditions [13].

The histological findings were paralleled by biochemical alterations observed in ApoA-IV-deficient mice exposed to HFD, including elevated serum ALT, triglyceride and insulin levels, which were not observed in WT mice. Elevated triglyceride and insulin concentrations are consistent with previous reports demonstrating impaired metabolic adaptation and insulin sensitivity in ApoA-IV-deficient mice [27]. The observed biochemical alterations may be considered alongside the transcriptomic changes identified in ApoA-IV-deficient mice. Elevated triglyceride concentrations were accompanied by altered expression of genes involved in lipid metabolism, including ApoC3 and cholesterol- and sterol-related pathways. Likewise, increased ALT levels and the persistent inflammatory phenotype may be consistent with alterations in inflammatory and stress–response pathways. Reduced expression of *Il6ra* in ApoA-IV-deficient mice exposed to HFD may indicate altered responsiveness to IL-6-mediated signaling, a pathway closely associated with hepatic inflammation, metabolic adaptation, and liver injury [50,51]. The repeated enrichment of cytochrome P450-related pathways may further reflect altered hepatic metabolic and detoxification responses, which could contribute to the biochemical phenotype observed in ApoA-IV-deficient mice, including elevated ALT levels and altered lipid metabolism [52,53]. In parallel, the robust induction of *Fgf21* in response to HFD in mice of both genotypes likely reflects activation of conserved adaptive mechanisms aimed at maintaining metabolic homeostasis under conditions of nutrient excess [54,55]. Although direct causal relationships cannot be established from the present data, the concordance between the biochemical and transcriptomic findings supports their biological relevance.

Although these changes were relatively modest, their concordance with the histological evidence of hepatic inflammation supports the biological relevance of the observed phenotype. These alterations occurred in the absence of overt steatosis or hepatocellular ballooning. Hepatocellular ballooning, a key histological feature required for the diagnosis of steatohepatitis (previously referred to as nonalcoholic steatohepatitis [NASH], currently metabolic dysfunction-associated steatohepatitis [MASH] under the MASLD nomenclature) [56,57], was not observed in any of the groups, indicating that the experimental model did not recapitulate a full steatohepatitis phenotype. Therefore, the transcriptomic, biochemical, and histological findings should be interpreted within the context of an early stage of hepatic adaptation and inflammation rather than established steatotic liver disease. The molecular and biochemical alterations identified in this study may represent adaptive responses that precede the development of more severe histopathological changes. Consequently, caution is required when extrapolating these findings to later stages of MASLD/MASH progression.

Under dietary challenge, the transcriptomic profiles of ApoA-IV-deficient and WT mice appeared more similar than under normal dietary conditions. Together with our previous observations [17], these findings are consistent with a contribution of host factors to early responses to dietary challenge; however, the relative contribution of host- and microbiome-dependent mechanisms was not directly addressed in the present study.

### Limitations

Several limitations of the present study should be acknowledged. First, the number of biological replicates available for some transcriptomic comparisons was limited, which may have reduced statistical power and constrained the detection of DEGs. In addition, transcriptomic profiling was performed using the Ion Proton platform, which has recognized limitations compared with newer sequencing technologies, particularly with respect to read characteristics and transcript detection sensitivity. This limitation should be considered when interpreting the relatively modest number of DEGs and the lack of clear global transcriptomic separation observed in PCA. Nevertheless, the repeated identification of similar biological pathways across independent comparisons supports the biological relevance of these findings and suggests that they are unlikely to represent random transcriptional variation. Second, the transcriptomic, biochemical, and histological changes identified in this study were observed in a model lacking extensive steatosis and hepatocellular ballooning. Therefore, the findings are best interpreted as reflecting early adaptive and inflammatory responses to dietary challenge rather than advanced steatotic liver disease and should not be directly extrapolated to later stages of disease progression. Third, the analysis was restricted to transcriptomic profiling, and the RNA-seq findings were not validated using an independent method such as RT-qPCR. Our study was intended to be exploratory and further studies integrating targeted gene expression analyses, proteomic, metabolomic, and functional approaches will be required to validate the biological significance of the identified pathways and their contribution to hepatic adaptation. Fourth, food intake was not monitored during the experimental period. Therefore, potential differences in energy consumption between groups could not be assessed, and their contribution to the observed metabolic and transcriptomic differences cannot be completely excluded. Additionally, only male mice were included in the study. Given known sex-dependent differences in metabolic and hepatic responses to HFD, future studies should include both sexes to assess the generalizability of the present findings. Finally, the use of a single experimental time point precludes assessment of the temporal dynamics of ApoA-IV-dependent responses to dietary challenge. Longitudinal studies will be required to determine how the observed molecular, biochemical, and histological changes evolve during disease progression.

## 5. Conclusions

Taken together, these findings indicate that ApoA-IV is associated with modulation of hepatic responses to dietary stress through specific metabolic and inflammatory pathways rather than through major transcriptomic or phenotypic alterations. ApoA-IV deficiency was associated with a more limited set of diet-related transcriptomic changes, although the biological significance of this observation requires further investigation. Notably, alterations in cholesterol- and sterol-related pathways, including the statin pathway, represented the most consistent genotype-associated transcriptomic signature and suggest a stable effect of ApoA-IV deficiency on hepatic cholesterol homeostasis. Within the context of the present model, the findings suggest that ApoA-IV may contribute to hepatic responses during the early stages of metabolic adaptation to dietary challenge, when molecular alterations can precede overt histopathological manifestations.

## Figures and Tables

**Table 1:** Histological characteristics of liver tissue samples according to the metabolic dysfunction-associated steatotic liver disease (MASLD) activity score.

		ApoA-IV-KO		WT	
	Score	ND	HFD	ND	HFD
		*n* = 8	*n* = 7	*n* = 8	*n* = 8
Lobular inflammation	0	1		6	3
	1	4	6		3
	2	2	1	2	2
	3	1			
	median (IQR)	1 (1–2)	1 (1–1)	0 (0–0.5)	1 (0–1.25)
Portal inflammation	0			1	
	1	1		2	2
	2	6	5	3	6
	3	1	2	2	
	median (IQR)	2 (2–2)	2 (2–2.5)	2 (1–2.25)	2 (1.75–2)
Ballooning	0	8	7	8	8
	1				
	2				
	median (IQR)	0 (0–0)	0 (0–0)	0 (0–0)	0 (0–0)
Steatosis	0	8	7	8	8
	1				
	2				
	3				
	median (IQR)	0 (0–0)	0 (0–0)	0 (0–0)	0 (0–0)
	0			4	4
	1	5	3	3	2
Fibrosis	2	3	4	1	2
	3				
	median (IQR)	1 (1–2)	2 (1–2)	0.5 (0–1)3	0.5 (0–1.25)

Number of animals and the median score calculated for the group were provided. Combined MASLD activity scoring system, based on [19,20]: lobular and portal inflammation (0, no foci; 1, <2 foci per 200× field; 2, 2–4 foci per 200× field; 3, >4 foci per 200× field); hepatocellular ballooning (0, none; 1, few cells; 2, many cells); steatosis (0, <5%; 1, 5–33%; 2, 34–66%; 3, >66%); fibrosis (0, none; 1, mild; 2, mild to moderate; 3, moderate to severe). ApoA-IV-KO, ApoA-IV knockout; HFD, high-fat diet; ND, normal diet; WT, wild-type. No comparison reached statistical significance (Mann–Whitney U-test with FDR correction).

**Table 2:** Serum biochemical parameters.

	ApoA-IV-KO		WT		*p*-Value
	ND	HFD	ND	HFD	
	*n* = 6	*n* = 6	*n* = 8	*n* = 6	
AST (IU/L)	62.0 (43.8–71.3)	55.5 (49.3–62.5)	76.0 (66.8–78.8)	96.5 (88.5–107.5)	0.02475 *
ALT (IU/L)	12.0 (11.3–15.8)	28.5 (23.5–34.3)	18.5 (16.0–25.0)	27.0 (25.3–31.8)	0.00318 *
ALP (IU/L)	143.0 (132.8–159.3)	107.5 (100.3–120.0)	144.5 (138.0–148.3)	122.5 (119.0–126.8)	0.0009767 *
T-CHOL (mg/dL)	53.0 (52.0–57.0)	97.0 (87.3–119.5)	54.0 (52.3–55.5)	129.5 (124.3–138.5)	0.0009561 *
HDL-C (mg/dL)	50.5 (47.5–54.3)	80.5 (70.8–92.5)	54.0 (33.5–57.0)	101.5 (96.3–105.3)	0.0003263 *
TRIG (mg/dL)	48.5 (42.0–50.3)	73.5 (63.5–86.5)	76.0 (66.5–89.3)	59.0 (56.3–65.5)	0.02541 *
GLU (mg/dL)	172.0 (152.5–176.5)	167.5 (135.3–196.8)	138.0 (133.3–145.8)	154.0 (145.0–163.0)	0.1121
INS (ng/mL)	0.99 (0.73–1.15)	5.43 (3.12–5.81)	2.42 (1.64–3.38)	1.59 (1.36–1.97)	0.002436 *

Median value and interquartile range were indicated. * Statistically significant in the Kruskal–Wallis rank-sum test; *p* < 0.05. AST, aspartate aminotransferase; ALT, alanine aminotransferase; ALP, alkaline phosphatase; T-CHOL, total cholesterol; HDL-C, high-density lipoprotein cholesterol; TRIG, triglyceride; GLU, glucose; INS, insulin; ApoA-IV-KO, ApoA-IV knockout; HFD, high-fat diet; ND, normal diet; WT, wild-type.

## Data Availability

The data that support the findings of this study were deposited at BioProject repository under accession number PRJNA1511580.

## Supplementary Materials

The following supporting information can be downloaded at: https://www.mdpi.com/article/10.3390/metabo16090695/s1, Figure S1: Representative images of the liver tissue presenting histological features identified in ApoA-IV-KO mice fed a high-fat diet (HFD). (A) Hematoxylin and eosin (H&E)-stained liver sections showing normal liver morphology, lobular inflammation, and portal inflammation. (B) Masson’s trichrome-stained liver sections showing absence of fibrosis and mild fibrosis, respectively. All images were acquired at 200× magnification; Figure S2: Pairwise comparisons of serum biochemical parameters: (A) AST, aspartate aminotransferase; (B) ALT, alanine aminotransferase; (C) T-CHOL, total cholesterol; (D) HDL-C, high-density lipoprotein cholesterol; (E) TRIG, triglyceride; (F) GLU, glucose; (G) ALP, alkaline phosphatase; (H) INS, insulin. Mann–Whitney U test significance: * FDR-adjusted *p* < 0.05; ** FDR-adjusted *p* < 0.01; Figure S3: Principal component analysis (PCA) based on transcriptome profiling of samples sorted by mouse strain and diet; Table S1: Differentially expressed genes (*p*_adj_ < 0.1) between ND-fed ApoA-IV-KO and WT mice; Table S2: Differentially expressed genes (*p*_adj_ < 0.1) between HFD-fed ApoA-IV-KO and WT mice; Table S3: Differentially expressed genes (*p*_adj_ < 0.1) between HFD-fed and ND-fed ApoA-IV-KO mice; Table S4: Differentially expressed genes (*p*_adj_ < 0.1) between HFD-fed and ND-fed WT mice; Table S5: Significantly (*p*_adj_ < 0.05) enriched functional categories in ClueGO analysis of 124 DEGs (*p*_adj_ < 0.1) differentiating between ND-fed ApoA-IV-KO and WT mice; Table S6: Significantly (*p*_adj_ < 0.05) enriched functional categories in ClueGO analysis of 32 DEGs (*p*_adj_ < 0.1) differentiating between HFD-fed ApoA-IV-KO and WT mice; Table S7: Significantly (*p*_adj_ < 0.05) enriched functional categories in ClueGO analysis of 20 DEGs (*p*_adj_ < 0.1) differentiating between HFD-fed and ND-fed ApoA-IV-KO mice; Table S8: Significantly (*p*_adj_ < 0.05) enriched functional categories in ClueGO analysis of 65 DEGs (*p*_adj_ < 0.1) differentiating between HFD-fed and ND-fed WT mice; Table S9: Shared and diet- or mouse strain-specific functional categories in ApoA-IV-KO vs. WT mice. Statistical significance: *p*_adj_ < 0.05; Table S10: Shared and diet- or mouse strain-specific functional categories in HFD-fed vs. ND-fed mice. Statistical significance: *p*_adj_ < 0.05.

## Abbreviations

The following abbreviations are used in this manuscript:

ApoA-IV	Apolipoprotein A-IV
HFD	High-fat diet
ND	Normal diet
KO	Knockout
WT	Wild-type
MASLD	Metabolic dysfunction-associated steatotic liver disease
HDL-C	High-density lipoprotein cholesterol
ALT	Alanine aminotransferase
AST	Aspartate aminotransferase
ALP	Alkaline phosphatase
RIN	RNA integrity number
WTS	Whole transcriptome sequencing
*P_adj_*	Adjusted *p*-value
PCA	Principal component analysis
DEG	Differentially expressed gene
GO	Gene Ontology
BP	Biological Process
MF	Molecular Function
CC	Cellular Component
ISP	Immune System Process
RNAseq	RNA sequencing
